# Identification of an elaborate NK-specific system regulating HLA-C expression

**DOI:** 10.1371/journal.pgen.1007163

**Published:** 2018-01-12

**Authors:** Hongchuan Li, Martin A. Ivarsson, Victoria E. Walker-Sperling, Jeff Subleski, Jenna K. Johnson, Paul W. Wright, Mary Carrington, Niklas K. Björkström, Daniel W. McVicar, Stephen K. Anderson

**Affiliations:** 1 Basic Science Program, Leidos Biomedical Research Inc., Frederick National Laboratory for Cancer Research, Frederick, MD, United States of America; 2 Center for Infectious Medicine, Department of Medicine Huddinge, Karolinska Institutet, Karolinska University Hospital, Stockholm, Sweden; 3 Cancer and Inflammation Program, Center for Cancer Research, National Cancer Institute, Frederick, MD, United States of America; 4 Ragon Institute of Massachusetts General Hospital, Massachusetts Institute of Technology and Harvard University, Cambridge, MA, United States of America; The Jackson Laboratory, UNITED STATES

## Abstract

The *HLA-C* gene appears to have evolved in higher primates to serve as a dominant source of ligands for the KIR2D family of inhibitory MHC class I receptors. The expression of NK cell-intrinsic MHC class I has been shown to regulate the murine Ly49 family of MHC class I receptors due to the interaction of these receptors with NK cell MHC in *cis*. However, *cis* interactions have not been demonstrated for the human KIR and HLA proteins. We report the discovery of an elaborate NK cell-specific system regulating HLA-C expression, indicating an important role for HLA-C in the development and function of NK cells. A large array of alternative transcripts with differences in intron/exon content are generated from an upstream NK-specific *HLA-C* promoter, and exon content varies between *HLA-C* alleles due to SNPs in splice donor/acceptor sites. Skipping of the first coding exon of *HLA-C* generates a subset of untranslatable mRNAs, and the proportion of untranslatable *HLA-C* mRNA decreases as NK cells mature, correlating with increased protein expression by mature NK cells. Polymorphism in a key Ets-binding site of the NK promoter has generated *HLA-C* alleles that lack significant promoter activity, resulting in reduced HLA-C expression and increased functional activity. The NK-intrinsic regulation of *HLA-C* thus represents a novel mechanism controlling the lytic activity of NK cells during development.

## Author summary

It has been proposed that the human *HLA-C* gene evolved in higher primates to serve as a ligand for the KIR family of inhibitory receptors for MHC class I that are expressed by natural killer (NK) cells and regulate their activity. NK cell potential is determined by the level of MHC class I on surrounding cells and on the NK cell itself. We have uncovered a highly complex system regulating HLA-C expression in NK cells. A NK-specific promoter produces a large array of differentially-spliced transcripts that vary in their ability to be translated into HLA-C protein. As NK cells differentiate and become more cytotoxic, the level of HLA-C expression increases, and this correlates with an increased abundance of translatable *HLA-C* mRNAs. A subset of HLA-C alleles have a promoter polymorphism that abrogates its activity, resulting in NK cells that are unable to upregulate HLA-C levels, and consequently, possess increased functional activity. Overall, our findings provide insight into the mechanisms of NK cell development, as well as a method to identify individuals with high NK activity, that may provide superior outcomes in hematopoietic stem cell transfer.

## Introduction

Natural Killer (NK) cells use two major receptor systems to detect alterations in the expression of MHC class I on potential target cells: the CD94:NKG2A receptor recognizing non-classical HLA-E, and the MHC class I receptors represented by Ly49 in the mouse and KIR in humans [1]. The recognition of HLA-E by NKG2A is dependent on the presentation of the MHC class I leader peptide, and thus surveys cells for the presence or absence of MHC class I expression in general. In contrast, each Ly49 or KIR is specific for a subset of MHC class I molecules, providing a more precise detection of alterations in the expression of individual MHC class I genes. Several studies have demonstrated a switch from NKG2A expression to Ly49/KIR expression as NK cells mature [2–4]. The measurement of HLA expression levels by mass spectroscopy of peripheral blood lymphocytes revealed that HLA-A/B/C levels are at least 25 times higher than that of HLA-E [5], suggesting that the level of inhibitory signaling by MHC class I receptors may increase as NK cells mature and switch from NKG2A recognition of HLA-E to KIR-mediated HLA binding.

The education of NK cells by MHC class I is currently an area of intensive research [6–8]. The interaction of inhibitory MHC class I receptors with their ligands has been shown to augment NK cell potential, leading to higher lytic activity and cytokine secretion. The dynamic nature of NK cell education has been revealed by transfer of NK cells into a novel MHC environment, leading to a change in their responsiveness [9–11]. A recent study of human NK cell education has indicated a role for NK cell-intrinsic expression of HLA in the tuning of NK cell activity, as silencing of HLA expression in primary NK cells reduced their function [12]. The role of the human *HLA-C* gene in NK cell education is of particular interest, as it appears to have developed primarily as a ligand for the KIR2D family of receptors [13,14]. Whereas only small subsets of *HLA-A* and *HLA-B* alleles possess KIR ligands, all *HLA-C* alleles are recognized. Furthermore, HLA-A or HLA-B cell surface expression levels are 13–18 times higher than HLA-C [5], consistent with a primary role of HLA-C in tuning NK cell responsiveness rather than presenting antigen to T cells. Evolutionary selection for an optimal level of KIR:HLA interaction is implied by the observed allelic variation of KIR cell surface expression levels and differences in ligand affinity of KIR alleles for HLA molecules [15]. Recent studies have also revealed variability in the level of cell surface expression of *HLA-C* alleles, indicating that variation in ligand levels may also be involved in the tuning of NK responsiveness [16,17].

In order to gain insight into the mechanisms underlying allele-specific differences in HLA-C expression, we conducted a detailed analysis of polymorphisms in predicted transcription factor (TF) binding sites in the 1.5 kb region upstream of the HLA-C coding region. Several TF sites were identified that possessed a disruptive single nucleotide polymorphism (SNP) associated with reduced promoter activity [18]. However, a SNP that disrupted a consensus Ets-binding site located approximately 1.3 kb upstream of the HLA-C start codon was not associated with altered promoter activity in the panel of cell lines studied. The detailed analysis of this region described in the current study reveals that the upstream Ets site is contained within an NK-specific promoter that produces translatable full-length *HLA-C* transcripts. The presence of these transcripts is associated with a higher level of HLA-C expression on NK cells. Disruption of the Ets site by a SNP in the HLA-C*02/*05/*07/*08 alleles results in the loss of NK-specific transcripts and decreased HLA-C expression. The analysis of NK cells from individuals homozygous for the Ets-disrupting SNP revealed that the loss of NK-specific *HLA-C* transcripts is associated with higher functional activity. The presence of NK-specific control elements in the *HLA-C* gene supports a central role for this gene in the development and regulation of NK cells.

## Results

### Identification of an NK-specific element in the *HLA-C* gene

**Fig 1:**
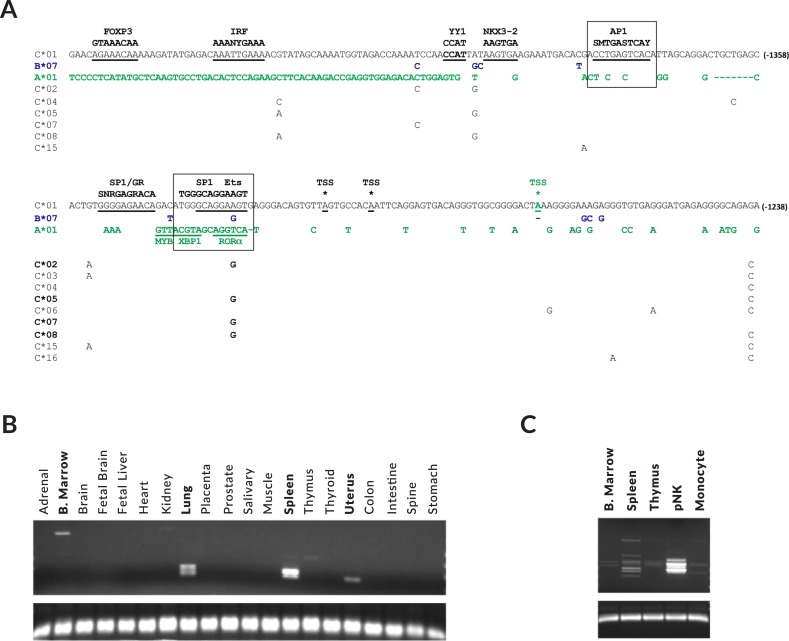
Identification of a promoter element in the -1300 region of the HLA genes. (A) The nucleotide sequence of the HLA-C*01:*02* gene from -1477 to -1238 upstream of the start codon is shown. Only nucleotide differences are shown in green for HLA-A*01, in blue for HLA-B*07, and in black for *HLA-C* alleles that differ from C*01:*02* in this region. Predicted TF-binding sites together with their consensus nucleotides are indicated in bold above the C*01:*02* sequence. The AP1 and combined SP1/Ets1-binding sites are boxed, and distinct TF sites present in the *HLA-A* gene are shown. The transcription start sites (TSS) of EST clones are indicated by asterisks in black for *HLA-C*, and in green for *HLA-A* (B) RT-PCR of a human tissue RNA panel with primers specific for upstream *HLA* transcripts is shown. Tissues of origin are listed for each lane, with PCR-positive lanes labeled in bold. The lower panel shows an amplicon generated with a primer set from *HLA-C* exons 7 and 8. (C) PCR of cDNA from purified peripheral blood NK cells (pNK), bone marrow (B. marrow), spleen, thymus, and purified monocytes using *HLA-C* specific exon-1a and exon 2 reverse primers. The lower panel shows an amplicon generated with a primer set from *HLA-C* exons 7 and 8.

A detailed analysis of the *HLA-C* gene region located ~1300 bp upstream of the start codon using the UCSC Genome Browser (http://genome.ucsc.edu/), revealed the presence of two spliced transcripts (GenBank numbers: DA932871, DA955942) that initiated 14 and 22 bp downstream of the polymorphic Ets element, suggesting the presence of a promoter in this region (Fig 1A). Both of these transcripts were obtained from a human spleen oligo-capped EST library, indicating that they represent true transcription start sites (TSS). The putative promoter region upstream of these TSS contains predicted AP1, SP1, and Ets elements that have previously been associated with the promoters of genes expressed by NK cells [19] (Fig 1A), suggesting that an NK-specific promotor may be present. Furthermore, a single nucleotide polymorphism in the Ets site of four *HLA-C* alleles (C*02, C*05, C*07, C*08) is predicted to abrogate Ets binding to this site and might affect promoter activity. The homologous region of the *HLA-A* gene has several nucleotide differences that disrupt the AP1-binding site, and the SP1/Ets element is replaced by binding sites for XBP1 and RORα (Fig 1A), indicating a distinct function for this region in the *HLA-A* gene. Transcripts from this region of *HLA-A* were observed in a macrophage EST library (GenBank numbers: BP306201, BP300425, BP297407), suggesting that a macrophage-specific promoter is present in this region of the *HLA-A* gene. This observation is consistent with the evolution of this region from supporting an antigen presentation function of HLA-A in macrophages to a role for HLA-C in NK cell function.

A panel of human tissue RNAs was tested for the presence of transcripts initiating from the putative upstream *HLA-C* promoter. Fig 1B shows the results of RT-PCR using a forward primer downstream of the observed initiation sites, but preceding a consensus splice donor site, and a reverse primer in exon 1. The strongest signal was found in spleen RNA, with weaker signals found in bone marrow, lung, and uterus, suggestive of NK-specific transcription. Only a very faint band is present in thymus, excluding T cells as a significant source of transcripts. Comparison of the level of PCR products generated from purified peripheral blood NK cell (pNK) cDNA with bone marrow, spleen, thymus, and purified monocyte cDNA, demonstrated that NK cells are the principal source of *HLA-C* upstream transcripts (Fig 1C). We will henceforth refer to the novel upstream *HLA-C* promoter as the NK-promoter (NK-Pro).

### Variable exon content and tissue-specific splicing patterns of NK-Pro transcripts

**Fig 2:**
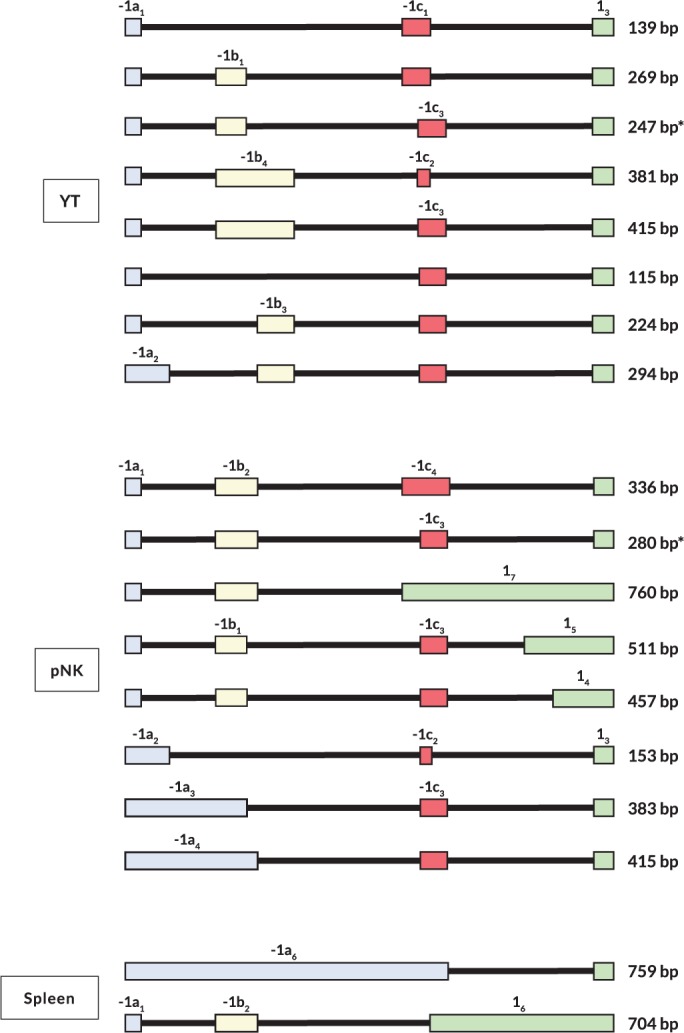
Variable 5´ UTRs detected in the human YT cell line, peripheral blood NK, and spleen. A schematic diagram of exon content observed by sequencing of PCR products generated using an exon -1a forward primer and an exon 1_3_ reverse primer located upstream of the HLA-C start codon. The relative size and position of exons in the 1.3 kb region upstream of the HLA-C start codon is shown. The size of each amplicon is listed on the right of each transcript. The asterisk indicates the most abundant splice form observed in YT or pNK cDNA. Only novel transcripts not found in YT cDNA are shown for pNK, and unique cDNAs not found in either YT or pNK are shown for spleen.

**Fig 3:**
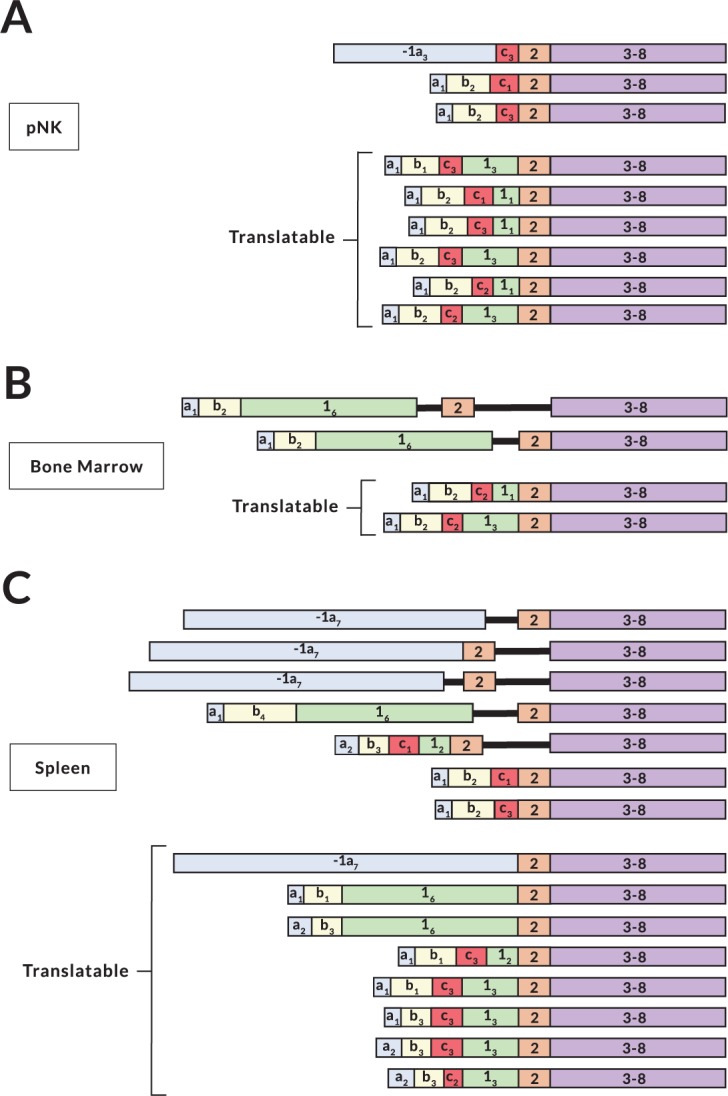
Full-length NK-Pro cDNAs identified in various human tissues. (A) Summary of full-length NK-Pro cDNAs cloned from peripheral blood NK cells (pNK) obtained from an individual homozygous for the HLA-C*06 allele. The lower group labeled (translatable) represents cDNAs that possess a complete HLA-C open reading frame. (B and C) Summary of clones identified from bone marrow or spleen cDNA generated from pooled human tissues.

**Fig 4:**
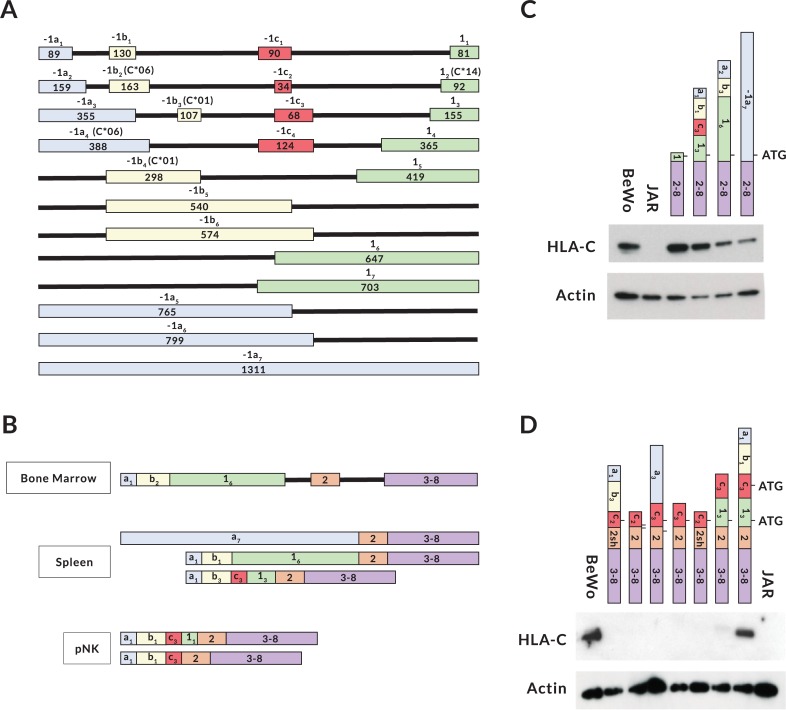
Variable exon content, tissue-specific splicing, and translatability of NK-Pro transcripts. (A) A schematic summarizing alternative exons detected in NK-Pro transcripts is shown. The three non-coding exons resulting from transcription initiating at NK-Pro are named as exons -1a, -1b, and -1c, with subscripts indicating the multiple variants of each exon. Allele-specific exons are indicated in parentheses. (C*01) indicates an exon found in the HLA-C*01 or C*04 alleles. (C*06) represents an exon from the HLA-C*06/*12 alleles. (C*14) is an exon found in the HLA-C*01, C*03, C*04, and C*14 alleles. The length of each exon is indicated. (B) A schematic of tissue-associated splice forms is shown for bone marrow, spleen, and peripheral blood NK cells (pNK). The most abundant transcripts are shown for bone marrow and pNK. The tissue-specific splice forms are shown for spleen. (C) Western blot of JAR cells transfected with expression constructs containing different lengths of 5´-UTR. Exon content of transfected cDNAs is shown schematically above each lane. HLA-C expressing BeWo cell lysate is shown on the left, followed by untransfected JAR cell lysate. (D) Western blot of JAR cells transfected with expression constructs lacking exon 1. The cDNA construct transfected is indicated schematically above each lane. BeWo cell lysate is shown in the left lane, and untransfected JAR cell lysate is on the right.

Interestingly, different patterns of amplified bands were observed in the tissues that produced NK-Pro transcripts (Fig 1B and 1C). Sequencing of the RT-PCR products from purified peripheral blood NK cells, spleen, and the YT human NK cell line revealed a large repertoire of alternatively spliced mRNAs (Fig 2). The highly variable nature of NK-Pro transcripts and the presence of a large 5´-UTR region containing competing initiation codons could provide an additional mechanism of modulating HLA-C protein expression, or it could reflect an enhancer/repressor function for the -1300 element, rather than a promoter capable of producing translatable *HLA-C* mRNAs. In order to assess the translatability of NK-Pro transcripts, full-length *HLA-C* cDNAs were generated from bone marrow, spleen, and peripheral blood NK cells from multiple donors possessing a variety of *HLA-C* alleles. Sequencing of the full-length products revealed additional alternative splicing events that could impact the translation of HLA-C (Fig 3). The most abundant bone marrow transcript retained intron 1, and was therefore untranslatable. Splice forms lacking exon 1 were observed in spleen and NK cell cDNA. Exon 1 contains the leader sequence of HLA-C. However, exon 2 contains an in-frame start codon, suggesting that an intracellular HLA-C molecule could be made. Fig 4A summarizes all of the exons observed. *HLA-C* transcripts initiating at the upstream promoter contain 1–3 additional non-coding exons that have been named -1a_1-7_, -1b_1-6_, and -1c_1-4_, with subscripts indicating differing exon sizes due to the use of alternative splice acceptors or donors for each exon. In addition, the size of the first HLA-C coding exon varies due to the presence of 7 alternative splice acceptors that can be used, so we have also named exon 1 isoforms as 1_1−7_. Interestingly, it appears that there has been selection for distinct exon variants in certain alleles, such as the observation of the -1b_2_ exon only in the HLA-C*06 and C*12 alleles, which is likely due to the presence of a SNP in these two alleles that generates a stronger splice donor consensus (G|GT versus A|GT in other alleles). The -1b_3_ and -1b_4_ exons are only found in cDNAs originating from the HLA-C*01 or *04 alleles, since the key G nucleotide of the consensus GT splice donor is only present in these alleles. Only the HLA-C*01, C*03, C*04, and C*14 alleles can generate exon 1_2_, due to a G to A nucleotide substitution that creates a splice acceptor site. The complex splicing patterns observed in *HLA-C* distal transcripts are not seen in *HLA-A*, which produces only one distal transcript containing two invariant untranslated 5´ exons (GenBank BP306201), indicating that modulation of the 5´-UTR structure has evolved specifically in the *HLA-C* gene.

Notably, there appears to be tissue-specific differences in exon usage of *HLA-C* NK-Pro transcripts (Figs 3 and 4B). Many of the NK-Pro transcripts in bone marrow retained intron 1, whereas a large 1.3 kb first exon (-1a_7_) was only observed in spleen, and most of the transcripts in peripheral blood NK cells contained a small 81 bp exon 1 (1_1_) and no intron retention was observed. The highly variable and tissue-specific splicing patterns observed for *HLA-C* NK-Pro transcripts suggests that HLA-C expression levels in NK cells from various tissues could be distinct.

### NK-Pro transcripts are translatable

In order to directly address the translatability of the alternatively spliced *HLA-C* mRNAs, full-length cDNAs were cloned into the pEF6 mammalian expression vector, transfected into the JAR trophoblast cell line that lacks HLA-C expression, and HLA-C protein levels were assayed by Western blot (Fig 4C and 4D). To evaluate the effect of 5´-UTRs of differing size on expression, a series of four constructs were tested, ranging from the full 1.3 kb UTR, to the minimal UTR generated by transcription from the proximal promoter. Fig 4C shows that the level of HLA-C protein produced decreased with increasing UTR size, suggesting that the variable splice forms with differing 5´-UTR sequences could “tune” the levels of HLA-C protein. Fig 4D demonstrates that exon 1 is required for HLA-C expression. None of the splice forms lacking exon 1 produced detectable HLA-C protein, indicating that the skipping of this exon results in an inefficiently translated mRNA or an unstable protein product. Furthermore, removal of exons -1a and -1b from a full-length NK-Pro transcript containing exon 1, so that the cDNA started at exon -1c, substantially decreased protein levels, suggesting that the mRNA secondary structure of the 5´-UTR of NK-Pro transcripts may prevent an ATG start codon in exon -1c from competing with the downstream HLA-C start codon. In addition, the cDNA containing the full 1.3 kb 5´-UTR was translatable (exon -1a_7_, Fig 4C), even though it contained multiple alternative 5´-ATG codons, further supporting a role for RNA secondary structure. The significant differences in *HLA-C* mRNA splicing in different tissues and at distinct stages of NK differentiation imply that changes in spliceosome function may play an important role in NK cell maturation or function.

### HLA-C levels increase and exon 1 skipping decreases as NK cells differentiate

**Fig 5:**
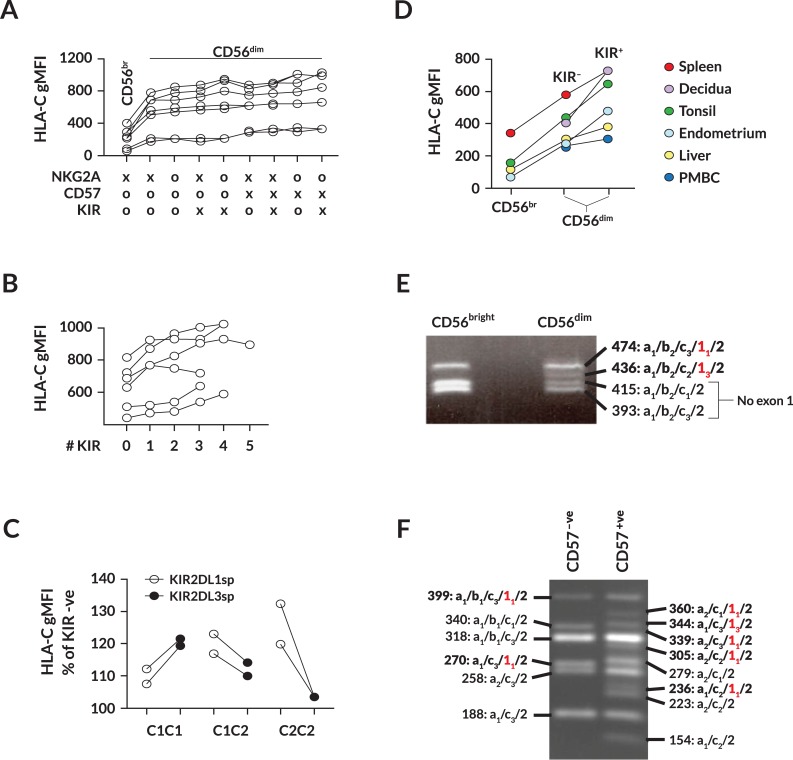
HLA-C expression levels increase and exon 1 skipping decreases as NK cells mature. (A) HLA-C geometric mean fluorescence intensity (gMFI) on NK cell subsets from seven PBMC donors. The presence (X) or absence (0) of NKG2A, CD57, or KIR (any combination of KIR2DL1/2/3/S1/S2/S4 or KIR3DL1) is indicated. The CD56^bright^ (CD56^br^) population is represented by a single phenotype, whereas CD56^dim^ NK cells contain eight distinct subsets. (B) HLA-C gMFI on CD56^dim^ NK cell subsets with differing numbers of co-expressed KIRs. PBMC from six donors were tested for the expression of the KIR2DL1/2/3/S1/S2/S4 or KIR3DL1 proteins on NK cell subsets. The number of co-expressed KIR (0–5) detected is plotted against the observed HLA-C level for each donor. (C) Comparison of HLA-C gMFI on NK cells expressing KIR2DL1 alone (KIR2DL1sp, negative for NKG2A, CD57 and other KIRs, open circles), versus KIR2DL3 single-positive NK cells (KIR2DL3sp, negative for NKG2A, CD57 and other KIRs, filled circles) in donors that possess only KIR2DL1 ligands (C2C2), KIR2DL3 ligands (C1C1) or both (C1C2). HLA-C expression is shown as a percentage relative to KIR-negative NK cells. Two donors of each *HLA-C* haplotype were tested. (D) Summary of HLA-C gMFI on CD56^bright^ versus CD56^dim^/KIR-negative (KIR-) and CD56^dim^/KIR-positive (KIR+) NK cells from various tissues. Decidual NK cells are shown as two points under KIR- and KIR+, since all cells in the decidua are CD56^bright^. (E) PCR using NK-Pro primers (5´exon -1a/3´exon 2) on cDNA from sorted CD56^bright^ versus CD56^dim^ NK cells from a donor homozygous for the HLA-C*06 allele. The size and exon content of each band is indicated on the right. Transcripts containing exon 1 are in bold, and red type indicates exon 1. (F) PCR of cDNA from sorted CD56dim/CD57-negative (CD57-ve) versus CD56^dim^/CD57-positive (CD57+ve) NK cells using the primers in (C), but from a donor containing the HLA-C*15/*16 alleles. The exon content of bands found in the CD57-negative subset are indicated on the left, and additional bands found in the CD57-positive cells are shown on the right.

The subset of untranslatable NK-Pro transcripts generated by skipping of exon 1 could potentially represent a mechanism to control HLA-C expression levels during NK development. In order to address the possibility of differential *HLA-C* mRNA splicing during NK development, HLA-C levels and the splicing patterns of NK-Pro transcripts were analyzed in NK subsets representing different stages of NK cell differentiation. Fig 5A shows the HLA-C expression levels in peripheral blood NK subsets from 7 individuals. There is a clear increase in HLA-C expression on the more mature CD56^dim^ NK cells relative to the less differentiated CD56^bright^ population in all subjects tested. As CD56^dim^ NK cells differentiate further, they acquire KIRs and CD57 [4]. This late-stage differentiation was also accompanied by an increase in HLA-C expression (Fig 5A and 5B). Analysis of educated NK cells (NK cells that express KIR molecules capable of recognizing self) revealed higher levels of HLA-C on educated NK cells as compared to non-educated or KIR^-ve^ NK cells (Fig 5C), indicating that high HLA-C expression occurs in highly functional, mature NK cells. FACS analysis of lymphocytes from various human tissues showed that increased expression of HLA-C is found on CD56^dim^ NK cells from multiple tissues, and the relative increase in HLA-C levels on KIR-expressing NK cells from tissues is similar to that seen in peripheral blood NK cells (Fig 5D). Furthermore, the level of HLA-C expression varies widely between tissues, which may reflect tissue-specific splicing (Fig 3) or differences in the activity of NK-Pro.

In order to determine if differential splicing of the NK-Pro transcript is associated with the changes in HLA-C expression observed, RT-PCR of NK-Pro transcripts was performed on RNA isolated from sorted CD56^bright^ versus CD56^dim^ peripheral blood NK cells (Fig 5E) or CD56^dim^/CD57-negative versus CD56^dim^/CD57-positive NK cells (Fig 5F). Fig 5E shows that the fraction of *HLA-C* NK-Pro mRNAs that contain exon 1 increases as cells progress from CD56^bright^ to CD56^dim^ NK. Fig 5F reveals an increase in the number of splice variants containing exon 1 in CD57-positive NK as compared to CD57-negative NK. Taken together, these results are consistent with a model whereby skipping of exon 1 results in the generation of non-productive transcripts, and represents a mechanism that prevents NK-Pro transcripts from generating increased HLA-C levels in immature NK cells. The comparison of splicing isoforms observed in Fig 5E and 5F also demonstrates the high degree of variability in splice forms observed with different *HLA-*C alleles. The donor analyzed in Fig 5E is homozygous for the HLA-C*06 allele, and a relatively simple splicing pattern is observed. In contrast, Fig 5F represents NK cells from a HLA-C*15/C*16 heterozygote, and a much greater number of distinct splice forms are observed, none of which are in common with the HLA-C*06 isoforms.

### Modulation of Ets and SP1 binding by a SNP in the Ets site

**Fig 6:**
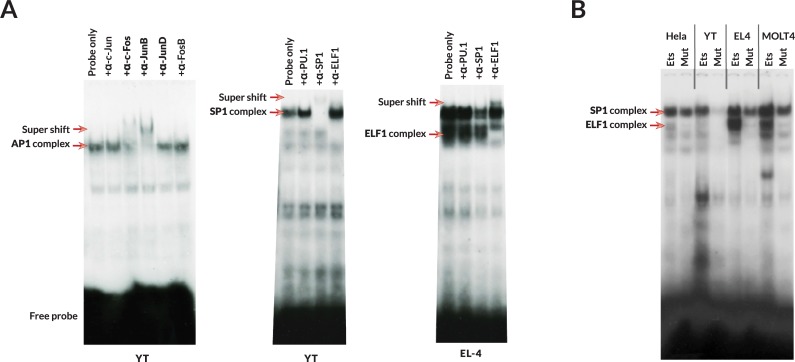
Binding of Fos/Jun and SP1/Elf to the NK-Pro elements. (A) EMSA performed with probes spanning either the AP1 or SP1/Ets sites of the *HLA-C* NK-Pro. The first panel shows results obtained using the AP1 probe with YT cell nuclear extracts. Inhibition of complex formation or supershift by various anti-Fos or Jun antibodies is shown. The middle panel shows results obtained with the SP1/Ets probe and YT extract, and the right panel shows complexes formed in EL4 extract. Inhibition of complex formation or supershift by anti-SP1 or Elf-1 antibodies is shown. (B) Comparison of TF binding to SP1/Ets probes containing either the wt (Ets) or mutant (Mut) Ets sites. Probe binding to nuclear extracts from HeLa, YT human NK cell, EL4 mouse T cell, and MOLT4 human T cell lines are shown.

In order to confirm the functionality of the predicted TF-binding sites in the NK-specific promoter and the effect of the Ets site SNP, an electromobility-shift assay (EMSA) was performed with oligonucleotides spanning the predicted AP1, SP1, and Ets TF-binding sites shown in Fig 1A. The AP1 site bound c-Fos and JunB proteins present in YT nuclear extract (Fig 6A). The combined SP1:Ets site bound both SP1 and the Ets family member Elf-1. The SNP in the NK-Pro Ets site was predicted to disrupt binding of Ets family members to this site, and EMSA with a probe containing the disruptive SNP demonstrated that the altered site had greatly diminished binding to both Elf-1 and SP1, most notably in the YT human NK cell line, indicating a cooperative interaction between these TFs (Fig 6B). A cooperative interaction between Ets family members and SP1 has been observed in many promoters [20,21], and tandem SP1/Ets sites have been identified in many genes, including the CD16 promoter [19]. The strong reduction in TF binding observed as a result of the A to G substitution in the Ets site (Fig 6B), suggests that transcriptional activity should be affected by this SNP found in the HLA-C*02/*05/*07/*08 alleles.

### Individuals lacking an intact Ets site have reduced NK-Pro transcript levels

**Fig 7:**
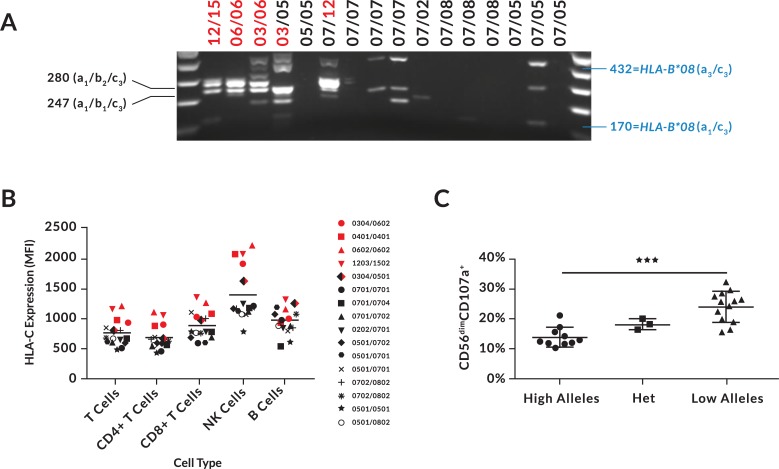
A disrupted Ets-binding site decreases NK-Pro transcription and HLA-C expression, resulting in higher lytic activity of NK cells. (A) RT-PCR performed with NK-Pro specific primers on purified NK cell RNA. The *HLA-C* genotype of each donor is listed above each lane. Red type indicates an allele that possesses an intact Ets site, whereas those in black type have a disrupted site. The size and exon content of the most abundant *HLA-C* transcripts is listed on the left, and two examples of HLA-B*08 transcripts detected are shown on the right. (B) Expression levels of HLA-C on peripheral blood lymphocytes is shown. Red symbols represent donors containing an intact Ets site, and black symbols are donors with disrupted Ets sites. (C) Summary of CD107a expression on CD56^dim^ NK cells after a 5-hour incubation with 721.221 target cells. Data shown are a summary of the results of four separate experiments testing 4–9 donors each time. *HLA-C* genotypes of individual donors are listed on the x-axis. (High) indicates donors homozygous for *HLA-C* alleles with an intact Ets site, (Low) indicates donors homozygous for a disrupted Ets site, and (Het) are individuals possessing one allele of each type. The line marked with (***) indicates p<0.001 for the difference between high and low donors using a Mann-Whitney ranked sum test.

The central role of an Ets site capable of binding Elf in conferring NK cell/T cell-specific promoter activity has been observed previously for the *MUNC4D* gene [22], and the in vitro analysis of NK-Pro indicated that an intact Ets site was required for TF binding to the SP1/Ets site. Therefore, we predicted a significant loss of NK-Pro transcription in *HLA-C* alleles with a disrupted Ets site. RT-PCR of the *HLA-C* NK-Pro transcript using RNA isolated from purified peripheral blood NK cells obtained from donors selected for the presence of alleles containing an intact or disrupted NK-Pro Ets site revealed that an intact Ets site was associated with the production of high levels of NK-specific transcripts (Fig 7A).

Individuals that were homozygous for any combination of the *HLA-C* alleles that lacked an intact Ets-binding site (HLA-C*02/05/07/08), had greatly reduced/absent *HLA-C* NK-Pro transcripts. Interestingly, in some of the individuals that lacked *HLA-C* NK-Pro transcripts, homologous transcripts derived from the HLA-B*08 gene were detected, suggesting the presence of NK-Pro activity in some *HLA-B* alleles. A Blast search of GenBank revealed that the HLA-B*14 allele contained an intact Ets site. However, HLA-B*08 and all other *HLA-B* alleles in GenBank contained the same SNP in the Ets site found in HLA-C*02/*05*/*07*/*08*. Interestingly, three of the donors shown in Fig 7A that did not produce any detectable transcripts had the HLA-B*14 allele, indicating that the Ets site is not associated with the production of distal transcripts in the *HLA-B* gene. This suggests that there are multiple nucleotide changes relative to *HLA-B* that are associated with NK-specific activity in the *HLA-C* gene in addition to the Ets site.

Fig 7B shows a FACS analysis of HLA-C expression by T, B, and NK cell subsets performed on peripheral blood from the same individuals tested for NK-Pro transcripts by RT-PCR. The presence of an intact Ets site in NK-Pro was associated with significantly higher expression of HLA-C by NK cells. A weaker effect of the Ets SNP was observed in T cells, indicating some activity of this element in T cells. However, there was no difference between intact versus disrupted Ets alleles with regard to HLA-C expression on B cells. It therefore appears that the -1300 element evolved in order to generate higher levels of HLA-C expression on NK cells. The functional effect of enhanced HLA-C expression by NK cells could be manifested in numerous ways. High HLA-C levels could protect NK cells from fratricide mediated by other NK [23], create a higher threshold for NK activation due to *cis* recognition, or it could lead to increased NK function as predicted by the observation that reduction of NK cell HLA expression can reduce NK activity [12].

### High HLA-C expression is associated with reduced NK activity

The functional consequences of high versus low HLA-C expression on NK cells was studied by comparing CD107a expression triggered by interaction of NK cells from high or low HLA-C expressing donors with 721.221 target cells. All donors possessed the *KIR2DL3*, *KIR2DL1*, and *KIR3DL1* genes present on the KIR-A haplotype to ensure that receptors for both HLA-C1/C2 and HLA-Bw4 were present. Both Bw4 and Bw6-homozygous individuals were tested in the assay in order to control for possible KIR3DL1/HLA-B effects. After a 5-hour incubation with target cells, NK cells from individuals with high HLA-C expression had significantly lower CD107a expression in the CD56^dim^ subset than donors with a disrupted *HLA-C* NK promoter and lower HLA-C expression (Fig 7C). The increased degranulation response in individuals with reduced HLA-C expression due to the absence of upstream transcripts implies an important role for *cis* expression of HLA-C in determining the functional activity of NK cells.

## Discussion

The presence of an NK-specific promoter coupled with an elaborate alternative splicing mechanism to control the translatability of *HLA-C* mRNA implies an important role for endogenous NK cell HLA-C protein in the development and/or function of NK cells. The increased NK activity we have observed in individuals unable to upregulate HLA-C levels on mature NK cells suggests that endogenous HLA-C plays a role in the tuning of NK cell function. These results are consistent with the evolution of the *HLA-C* gene to function as a ligand for the KIR family of MHC class I receptors expressed by human NK cells. The change in RNA splicing patterns and the increase in HLA-C expression levels as NK cells mature provides additional evidence for a role of HLA-C in controlling human NK cell function. It is remarkable that alternative RNA splicing generates both on/off (intron retention and exon skipping) and rheostat-like (variation in 5´-UTR length) control of HLA-C expression in NK cells, consistent with endogenous HLA-C levels playing an important role in NK function or differentiation. There is a considerable body of evidence demonstrating an association of increased NK cell activity with the presence of KIR that recognize self HLA (NK education). However, this correlation has been attributed to the recognition of HLA on target cells. MHC class I on the NK cell surface has been shown to control NK activity in mouse NK cells, and this is explained by the ability of Ly49 to recognize class I MHC in cis due to the presence of a flexible stalk in the Ly49 proteins. Although *cis* interactions with MHC have been demonstrated for the Ly49 receptors [24], and *cis* interaction is required for murine NK cell licensing [25], there has been no direct evidence of *cis* interaction of KIR with HLA. Since KIR do not have a flexible stalk, it is believed that cis interaction of KIR with HLA on the cell surface does not occur. It may be possible, however, for inhibitory signaling to occur in endosomes if both KIR and HLA are present. Vesicles containing target cell HLA-C have been observed in KIR2DL1-expressing NK cells, indicating acquisition and internalization of ligand by KIR [26]. It is therefore possible that high levels of endogenous NK cell HLA-C may contribute to inhibitory signaling in these endosomes. The results presented here, together with the previous observation that modulation of HLA expression in NK cells suppresses their activity [12], strongly suggests that KIR:HLA interactions are occurring in human NK cells. The maturation of NK cells from a CD56^bright^ to a CD56^dim^ phenotype is associated with the acquisition of lytic activity [27]. HLA-C levels increase in CD56^dim^ NK cells as well as mature CD57-positive NK cells, and this upregulation is associated with higher levels of translatable NK-Pro *HLA-C* transcripts due to increased inclusion of exon 1. Therefore, it appears that HLA-C levels increase when the NK cell acquires lytic activity, or alternatively the acquisition of lytic activity is triggered by increased HLA-C levels.

**Fig 8:**
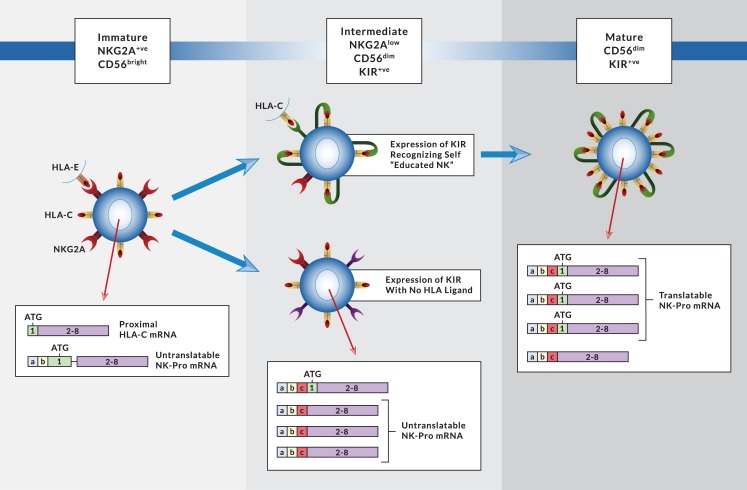
Changes in NK-Pro transcript structure during NK differentiation. The *HLA-C* transcript profile associated with distinct stages of NK cell differentiation is shown. CD56^bright^, immature NK cells express HLA-C primarily from proximal transcripts, as the majority of NK-Pro mRNAs present at this stage are untranslatable. In the intermediate stage, CD56^dim^ NK cells turn on KIR selectively. A large proportion of the NK-Pro transcripts present at the intermediate stage lack exon 1 and are untranslatable. If a KIR recognizing self HLA is present, the NK cell becomes educated, and develops into a fully mature, highly lytic effector cell. The mature NK cell has increased levels of translatable NK-Pro transcripts, and increased cell surface expression of HLA-C. Inhibition of the NK cell by endogenous HLA-C is indicated by KIR receptor binding to HLA-C in cis, however this would be predicted to occur in endosomes rather than on the cell surface.

At first glance, the increased functional activity of NK cells with reduced HLA-C due to the lack of NK-Pro transcripts seems to be at odds with the observed increase in HLA-C levels on NK cells as they become more mature and functionally active. However, these results are consistent with a model wherein the upregulation of HLA-C in mature NK cells occurs to regulate their function rather than having a direct effect on education. *KIR* gene expression is activated by a stochastic mechanism in developing NK cells, and there is sequential receptor acquisition until a sufficient inhibitory signal is achieved, which results in a fully functional, educated NK cell [28]. The observed increase in HLA-C expression that occurs with increasing numbers of expressed KIR genes in an NK cell suggests that increased HLA-C levels are a result of NK cell education, rather than driving it. In order for upregulated levels of NK cell HLA-C to play a role in NK education, it would have to occur in the uneducated NK population. Furthermore, it has been shown that differing levels of HLA-C have no effect on the licensing/education of NK cells [29]. The higher levels of HLA-C observed on educated versus uneducated NK cells, further supports upregulation of HLA-C on NK cells as a product of NK education rather than a cause. Fig 8 shows a schematic detailing the changes in NK-Pro mRNA structure and HLA-C expression as NK cells differentiate. The immature CD56^bright^, NKG2A^+ve^, KIR^-ve^ cells have low levels of HLA-C produced primarily from proximal promoter transcripts, since NK-Pro cDNAs in immature cells are largely untranslatable. At the intermediate stage of differentiation, KIR expression is stochastically activated in CD56^dim^ cells, and the presence of a ligand for the expressed KIR produces an educated NK cell. This leads to upregulated HLA-C in the mature NK cell due to an increase in the level of translatable *HLA-C* NK-Pro mRNAs. Increased levels of HLA-C in the mature NK cell are predicted to control lytic activity due to cis inhibitory signaling.

The tissue-specific differences in mRNA splicing of the NK-Pro transcript and the differences in HLA-C protein expression on NK cells from various tissues that we have observed could represent a tuning mechanism that regulates the responsiveness of NK cells in certain tissues. It will therefore be of interest to examine if the observed differences in HLA-C levels on NK cells from different tissues correlates with their ability to recognize and lyse targets with reduced HLA-C expression. The increased activity of NK cells with low HLA-C expression due to the presence of an inactivating SNP in NK-Pro suggests that the threshold of NK cell activation is lower, which might also produce a state of decreased lytic potential over time due to more frequent degranulation. Conversely, an increased accumulation of granules, and thus lytic potential would be predicted to occur in NK cells with a high level of HLA-C. Testing the serial-killing activity of high versus low HLA-C expressing NK cells could address this possibility.

Allele-specific differences in 5´-UTR exon content also imply selection for an optimal level of HLA-C expression in NK cells, perhaps in conjunction with allelic differences in KIR-binding affinity, in order to achieve an appropriate level of inhibitory signaling. A detailed examination of NK cell HLA-C levels in individuals that are homozygous for HLA-C alleles with distinct splicing patterns and comparing expression levels with the affinity of the allele for the corresponding KIR would be required to investigate this.

The existence of *HLA-C* alleles of both C1 (C*07/C*08) and C2 (C*02/C*05) supratypes with an inactivated NK-specific promoter suggests that there may be circumstances wherein the absence of upregulated expression of NK cell HLA-C would be beneficial. It will be of interest to determine whether there are any associations of NK-Pro deficient *HLA-C* alleles with clinical outcomes in infectious disease or bone marrow transplantation.

## Materials and methods

### Cell lines

HeLa, EL-4, MOLT-4, and the JAR/BeWo human trophoblast cell lines were obtained from ATCC (Manassas, VA, USA) and grown in the recommended media. YT cells were cultured in RPMI 1640 media containing 10% fetal bovine serum, 100 U/ml penicillin, 100 U/ml streptomycin, sodium pyruvate and L-glutamine.

### Donors and NK cell isolation

Healthy volunteers were recruited through the NCI-Frederick Research Donor Program (http://ncifrederick.cancer.gov/programs/science/rdp/default.aspx). The *KIR* and *HLA* genotype of each donor was determined as previously described [30]. NK cells were separated from the peripheral blood of healthy donors by Histopaque (Sigma-Aldrich, St Louis, MO, USA) gradient centrifugation using the RosetteSep Human NK Cell Enrichment Cocktail (STEMCELL Technologies, Vancouver, BC, Canada). Human tissue samples were obtained from surgically removed tissue (liver, spleen, endometrium, decidua, tonsil) at the Karolinska University Hospital, Stockholm, Sweden. Written and oral informed consent was obtained from all patients, and the study was approved by the Regional Ethics Review Board, Stockholm, Sweden (approval numbers: 2017-1659-32, 2013/2285-31/3, 2006/229-31/3, 2013/1324-31/2, 2017-649-31/1). Lymphocytes from liver, decidua, and endometrium were isolated using enzymatic digestion as previously described [31]. Tonsil and spleen were mechanically dissociated using scalpels followed by filtration. Mononuclear cells were obtained by density centrifugation using Histopaque (Sigma-Aldrich).

### RT-PCR of NK-Pro transcripts

Total RNA from 20 human tissues (Human total RNA master panel II) was obtained from Clontech (Mountain View, CA, USA). Total RNA from purified NK cells or the YT cell line was isolated from 1–5 x 10^6^ cells with the RNeasy kit (Qiagen, Valencia, CA, USA). A cDNA synthesis reaction was carried out using Random Hexamer primer, Taqman Reverse Transcription Reagents kit (Applied Biosystems, Foster City, CA, USA) according to the manufacturer’s instructions. A forward primer in exon -1a of the NK-Pro transcript (5´-AGAAGGGCTGGAGAAGCAGGAG-3´) was used together with an exon 1_3_ reverse primer upstream of the major *HLA-C* TSS (5´-GGACTGCGGAGACGCTGATTGG-3´) for the initial detection of NK-Pro transcripts. Additional *HLA-C* specific exon-1a forward (5´-GGGATGAGAGGGGCAGASAG-3´) and exon 2 reverse (5´-GTGCCTGGCGCTTGTASTTC-3´) primers were used to confirm the NK-specificity of *HLA-C* transcripts (Fig 1C). For full-length transcripts, an alternative 3´ primer located immediately following the HLA-C stop codon was used (5´-GTCCCACACAGGCAGCTGTCTC-3´). To assay the level of exon 1 skipping, an exon 2 reverse primer was used (5´-GAACTGCGTGTCGTCCACGTAG-3´). PCR products were cloned into the pCR2.1-topo vector (Invitrogen, Carlsbad, CA, USA) and sequenced. The sequences of the alternatively spliced NK-Pro transcripts have been deposited in GenBank, and can be found under accession numbers MF536989-MF536999 for the peripheral blood NK cell cDNAs, and accession numbers MF563479-MF563493 for the spleen and bone marrow cDNAs.

### Western blotting of *HLA-C* transfectants

Full-length *HLA-C* cDNAs with varying 5´-UTR lengths were cloned into the pEF6/V5-His TOPO-TA vector (Thermo Fisher Scientific, Waltham, MA, USA) and verified by sequencing. 1 ug of each construct was transfected into the human JAR trophoblast cell line using HilyMax Transfection Reagent (Dojindo Molecular Technologies Inc., Rockville, MD, USA). Cells were harvested with Nonidet-P40 (NP-40) lysis buffer (1% NP-40, 50 mM Tris-HCl, pH 8.0, 150 mM NaCl) supplemented with complete mini protease inhibitor cocktail tablets (Roche Diagnostics, Indianapolis, IN, USA), and protein concentrations were determined using a Nanodrop 2000 spectrophotometer (Thermo Fisher Scientific). Equal amounts of total protein were separated using sodium dodecyl sulfate-PAGE (SDS-PAGE) on 4–12% Tris-Glycine gels (Invitrogen) and transferred to Immobilon-P membrane (Sigma-Aldrich). Membranes were blocked for one hour in 5% milk solution in PBST (Phosphate Buffered Saline, pH 7.4, 0.1% Tween 20) and subsequently probed for 1.5 hours with an anti-HLA-C antibody (Abcam, Cambridge, MA, USA; ab126722) diluted 1:5000 in 5% milk PBST solution. Blots were washed 4 times for 5 minutes each with PBST and then were incubated for 20 minutes with an anti-rabbit HRP-linked IgG antibody (Cell Signaling Technology, Danvers, MA, USA) diluted 1:20,000 in 5% milk PBST solution. Blots were washed 4 times for 5 minutes with PBST and proteins were visualized using Amersham ECL Western blotting detection reagents (GE Healthcare, Pittsburgh, PA, USA). Normalization for protein loading was accomplished by first stripping blots with SDS stripping buffer for 10 minutes and then washing 3 times with PBS, pH7.4 for 5 minutes. Blots were then probed for 1 hour with a monoclonal anti-β-Actin antibody (Sigma-Aldrich A2228) at a concentration of 1 μg/ml followed by an anti-mouse HRP linked IgG antibody (Cell Signaling Technology) for 20 minutes diluted 1:20,000 in 5% milk PBST. Levels of β-Actin were visualized using Amersham ECL Western detection (GE healthcare).

### Electrophoretic mobility-shift assays (EMSA)

Nuclear extracts were prepared from cell lines using the CellLytic NuCLEAR extraction kit (Sigma-Aldrich). Protein concentration was measured with a Bio-Rad protein assay, and samples were stored at −70°C until use. Double-stranded DNA oligonucleotide probes containing either the AP1 site (5´-GACACGACC**TGAGTCA**CATTAGC-3´) or the the combined SP1/Ets site (5´-GACAT**GGGCAGGAAGT**GAGGGAC-3´) were synthesized (IDT, Newark, NJ, USA). Probes were labeled with α-[32P]deoxycytidine triphosphate (3000 Ci/mmol; PerkinElmer, Waltham, MA, USA) by fill-in using the Klenow fragment of DNA polymerase I (Invitrogen). 32P-labeled double-stranded oligonucleotides were purified using mini Quick Spin Oligo Columns (Roche). DNA–protein binding reactions were performed in a 10-μl mixture containing 5 μg nuclear protein and 1 μg poly[dI-dC] (Sigma-Aldrich) in 4% glycerol, 1 mM MgCl2, 0.5 mM EDTA, 0.5 mM DTT, 50 mM NaCl, 10 mM Tris-HCl (pH 7.5). Nuclear extracts were incubated with 1 μl 32P-labeled oligonucleotide probe (10,000 cpm) at room temperature for 20 min and then loaded on a 5% polyacrylamide gel (37:5:1). Electrophoresis was performed in 0.5x TBE for 2 h at 130 V, and the gel was visualized by autoradiography.

### HLA-C expression on peripheral blood lymphocytes and tissue-derived NK cells

Lymphocytes isolated from whole blood via Ficoll-Paque Plus gradient centrifugation (GE Healthcare) from 16 healthy volunteers were stained extracellularly with antibodies to determine expression of HLA-C on various subsets. The antibodies used were: CD3 [clone UCHT1] BV605, CD19 [clone HIB19] PE-Cy7, CD56 [clone HCD56] BV711 (BioLegend, San Diego, CA, USA); CD4 [clone SK3] PE-Cy7, CD8 [clone RPA-T8] APC (BD Biosciences, Franklin Lakes, NJ, USA); HLA-C [clone DT9] conjugated to AlexaFluor488. Data from the samples collected on a BD LSRFortessa flow cytometer were analyzed using FlowJo v10.1. Mononuclear cells from additional blood donors and from indicated tissues were analyzed by flow cytometry at the Karolinska Institutet, Stockholm, Sweden. The following additional antibodies were used: CD3 [clone UCHT1] PE-Cy5 (Beckman Coulter, Brea, CA, USA); CD19 BV510 (Biolegend); NKG2A [clone Z199] BB515, CD56 [clone NCAM16.2] BUV737, CD57 [clone NK-1] BV605, HLA-C [clone DT9] PE, KIR2DL2/3/S2 [clone CH-L] BUV395 (BD Biosciences); KIR2DL1/S1 [clone EB6] PC-5.5 (Beckman Coulter); KIR3DL1 [clone DX9] BV421 (Biolegend); KIR2DS4 [clone REA860] PE-Vio615 (Miltenyi Biotec, Cambridge, MA, USA). Live/Dead marker Aqua (Thermo Fisher Scientific) was also used. Samples were acquired on a 5 laser BD LSRFortessa flow cytometer and analyzed using FlowJo v10.1.

### CD107a NK cell degranulation assay

The frequency of degranulated human NK cells was quantitated by flow cytometric detection of cell surface CD107a. Purified donor human NK cells (10^5^) were co-cultured with 721.221 tumor cells in a round bottom 96-well plate at an effector target ratio of 1:1 in RPMI 1640 supplemented with 10% FBS, 10 mM Hepes, 2 mM L-glutamine, 0.1 mM nonessential amino acids, 1 mM sodium pyruvate, Penicillin 100 U/ml, Streptomycin 100 ug/ml, and 100 IU/ml recombinant human interleukin 2 (Tecin; Teceleukin, obtained from the Biological Resources Branch of the National Cancer Institute). Anti-human CD107a PE or BV421 (Biolegend) was added at 3 ul to 200 ul of the co-culture and the mixture was gently mixed by pipetting and then spun down at 50 g for 5 minutes and incubated for 1 h at 37°C in 5% CO_2_ after which Golgi-Stop (BD Biosciences) was added and cells were incubated for an additional 4 h at 37°C in 5% CO_2_. Cells were washed with sorter buffer (HBSS containing 0.5% BSA, 1mM EDTA, 25mM Hepes, 0.05% sodium azide) and Fc receptors were blocked with human FcR Blocking Reagent (Miltenyi Biotec) for 5 minutes and then then stained with anti-human CD56 PE or APC (clone HCD56, Biolegend) for 10 minutes. Stained cells were washed twice with sorter buffer and fixed with BD Cytofix (BD Biosciences) for 20 minutes. Cells were washed twice with sorter buffer and flow cytometric analysis was performed on a LSRFortessa instrument (BD Biosciences). Degranulated NK cells were analyzed using FlowJo software by gating on single cells (FSC-H x FSC-A) in the lymphocyte gate and then the frequency of CD56^dim^ CD107a^+^ cells was quantitated.
